# Bmi-1 Absence Causes Premature Brain Degeneration

**DOI:** 10.1371/journal.pone.0032015

**Published:** 2012-02-20

**Authors:** Guangliang Cao, Minxia Gu, Min Zhu, Junying Gao, Ying Yin, Charles Marshall, Ming Xiao, Jiong Ding, Dengshun Miao

**Affiliations:** 1 Jiangsu Province Key Laboratory of Neurodegeneration, Department of Anatomy, Nanjing Medical University, Nanjing, Jiangsu, People's Republic of China; 2 The Research Center for Bone and Stem Cells, Department of Anatomy, Nanjing Medical University, Nanjing, Jiangsu, People's Republic of China; 3 Department of Rehabilitation Sciences, University of Kentucky Center For Excellence in Rural Health, Hazard, Kentucky, United States of America; National Institute of Health, United States of America

## Abstract

Bmi-1, a polycomb transcriptional repressor, is implicated in cell cycle regulation and cell senescence. Its absence results in generalized astrogliosis and epilepsy during the postnatal development, but the underlying mechanisms are poorly understood. Here, we demonstrate the occurrence of oxidative stress in the brain of four-week-old Bmi-1 null mice. The mice showed various hallmarks of neurodegeneration including synaptic loss, axonal demyelination, reactive gliosis and brain mitochondrial damage. Moreover, astroglial glutamate transporters and glutamine synthetase decreased in the Bmi-1 null hippocampus, which might contribute to the sporadic epileptic-like seizures in these mice. These results indicate that Bmi-1 is required for maintaining endogenous antioxidant defenses in the brain, and its absence subsequently causes premature brain degeneration.

## Introduction

Bmi-1 is a member of the Polycomb family of transcriptional repressors. It is implicated in cell cycle regulation and cell senescence by repression of Ink4a/Arf locus, encoding cell cycle regulators p16Ink4a and p19Arf [1], [2], [3], [4], [5], [6]. Furthermore, a recent study has revealed that Bmi-1 regulates mitochondrial function, reactive oxygen species (ROS) levels and activation of the DNA damage response pathway [7].

In the central nervous system (CNS), Bmi-1 protein is localized within neural stem cells (NSCs), as well as mature neurons and astrocytes, indicating a critical role in brain development and maintenance [8], [9], [10]. Several *in vitro* and *vivo* studies have demonstrated that Bmi-1 regulates NSC proliferation and self-renewal [11], [12], [13], [14], [15]. Bmi-1 null (Bmi-1^−/−^) mice exhibit smaller brains with profound defects in cerebellar growth after 2 weeks of age [16]. Moreover, Bmi-1^−/−^ mice develop generalized astrogliosis, progressive ataxia and epilepsy in the first month after birth, but the underlying mechanisms are poorly understood [10], [16], [17].

The brain is particularly vulnerable to oxidative stress due to its high metabolic rate, high lipid content and limited antioxidant defenses [18], [19], [20]. Therefore, we propose that absence of Bmi-1 causes brain oxidative stress, which may be a primary mechanism for premature neurodegeneration. In addition, it is known that astrocytes are responsible for modulation of neurotransmitter release and synchronization of neuronal firing [21], [22]. Reactive astrocytes accompanied with alterations in glutamate transporters and glutamine synthetase have been observed in the brains of patients with temporal lobe epilepsy and animal models of epilepsy [23], [24]. Thus, an additional aim of the present study is to determine whether there are altered expressions of markers for glutamate uptake and conversion in the brain of Bmi-1^−/−^ mice.

## Results

### Oxidative stress in Bmi-1 null brain

**Figure 1:**
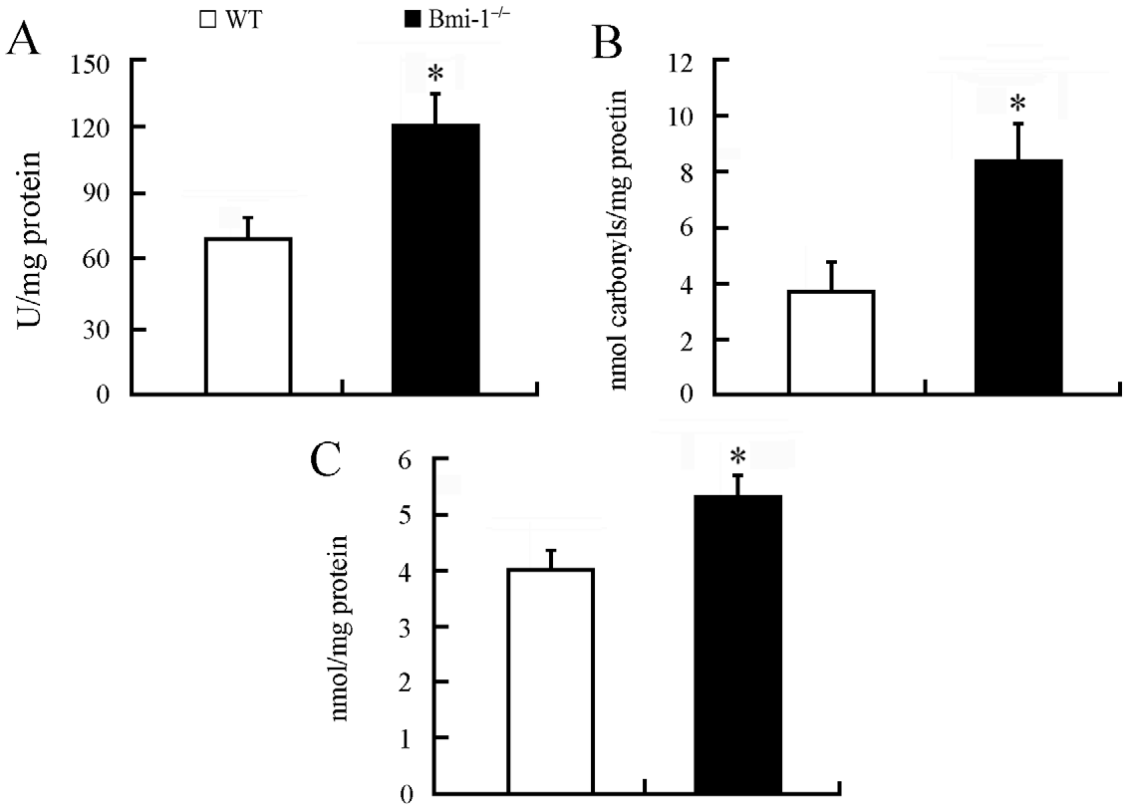
Brain oxidative stress in 4-week-old Bmi-1^−/−^ mice. Brain tissues from Bmi-1^−/−^ mice showed higher levels of hydroxyl radical (120.82±14.72 vs. 69.41±10.04 U/mg protein; A), protein carbonyl (8.37±1.41 vs. 3.86±1.09 nmol/mg protein; B) and malondialdehyde (5.34±0.45 vs. 4.03±0.41 nmol/mg protein; C) than those from WT controls. Five mice per genotype and 3 independent experiments for each homogenized brain sample. Data are expressed as mean ± SEM. *P<0.05 vs. WT mice.

Bmi-1 is implicated in maintaining the redox homeostasis in bone marrow cells and freshly isolated thymocytes [7]. To demonstrate whether deletion of Bmi-1 results in brain oxidative stress, we collected brain homogenate samples of 4-week-old Bmi-1^−/−^ mice and wild-type (WT) controls and measured hydroxyl radical, protein carbonyl and malondialdehyde levels, markers of oxidative damage in DNA, proteins and lipids, respectively. Our results showed that each oxidative marker increased in the Bmi-1 null brain compared with WT control (Figure 1). Further experiments revealed a significant elevation of hydroxyl radical levels but not protein carbonyl and malondialdehyde in the brain of 2-week-old Bmi-1^−/−^ mice (Figure S1). These results indicate that oxidative DNA damage is an early event in the pathogenesis of neurodegeneration.

### Impairment of neuronal elements in Bmi-1 null hippocampus

**Figure 2:**
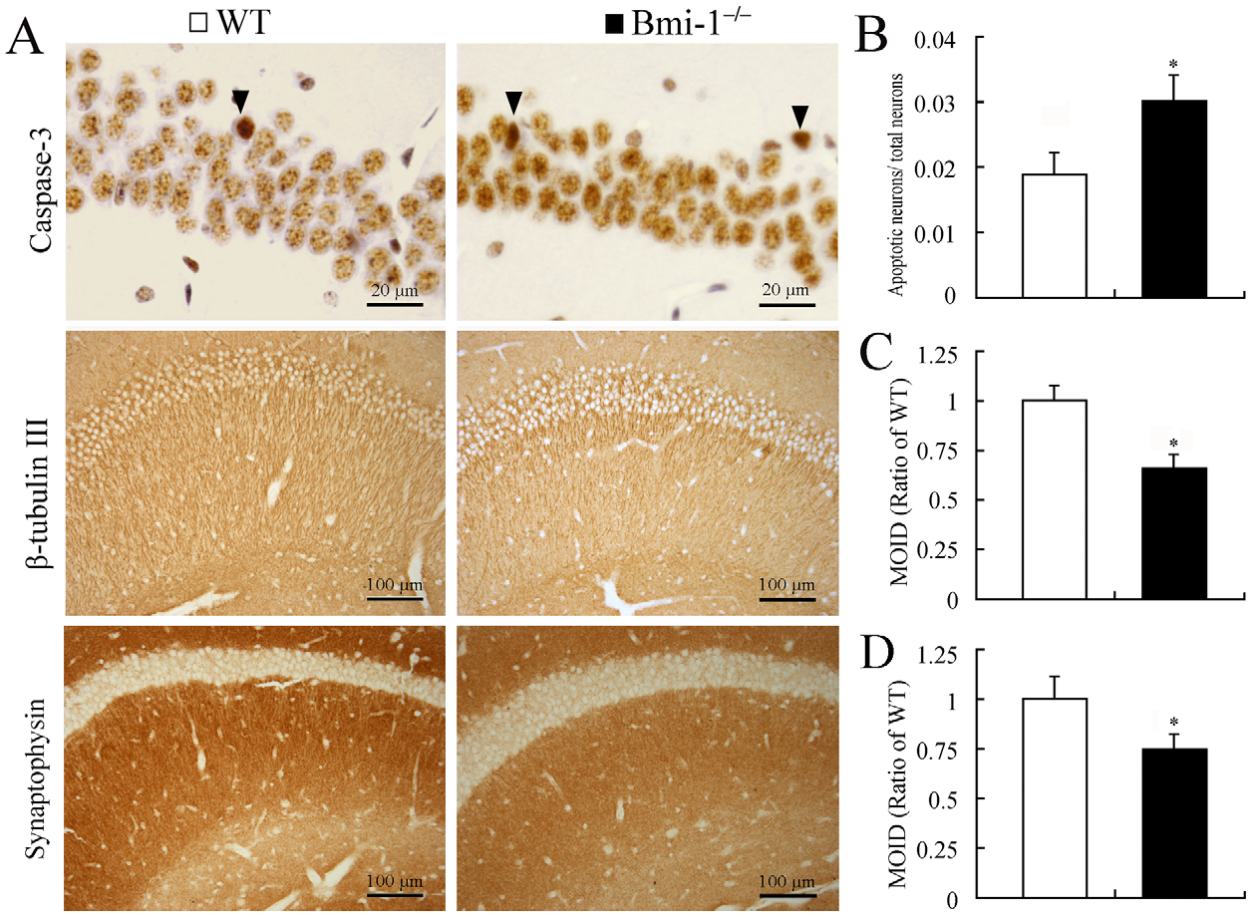
Degeneration of neuronal elements in the hippocampus of 4-week-old Bmi-1^−/−^ mice. (**A**) Immunostaining for caspase-3, β-tubulin III and synaptophysin. Arrowheads showing apoptotic pyramidal neurons which contained dense caspase-3 immunostaining. Immunoreactivities of β-tubulin III and synaptophysin were decreased in the Bmi-1 null hippocampus. (**B–D**) There were a higher ratio of apoptotic neurons to total neurons (0.031±0.004 vs. 0.019±0.003; B) and lower mean integrated optical densities (MIOD) of immunostainings for β-tubulin III (0.66±0.07 vs. 1±0.08; C) and synaptophysin (0.75±0.08 vs. 1±0.12; D) in the hippocampus of Bmi-1^−/−^ mice compared with WT controls. Five mice per genotype and 3 sections per mouse. Data are expressed as mean ± SEM. *P<0.05 vs. WT mice.

Hippocampal neurons are highly sensitive to various oxidative insults [18]. We addressed the influence of Bmi-1 deletion on the degeneration of hippocampal neurons. We performed immunostaining for caspase-3 to determine apoptotic neurons, β-tubulin III for neurites, and anti-synaptophysin to determine the presynaptic terminals (Figure 2A). Four-week-old Bmi-1^−/−^ mice showed a slightly higher ratio of apoptotic neurons to total neurons in the pyramidal layer compared with WT littermates (Figure 2B). Conversely, immunoreactive levels of β-tubulin III and synaptophysin were dramatically decreased in the Bmi-1 null hippocampus (Figure 2C–D).

**Figure 3:**
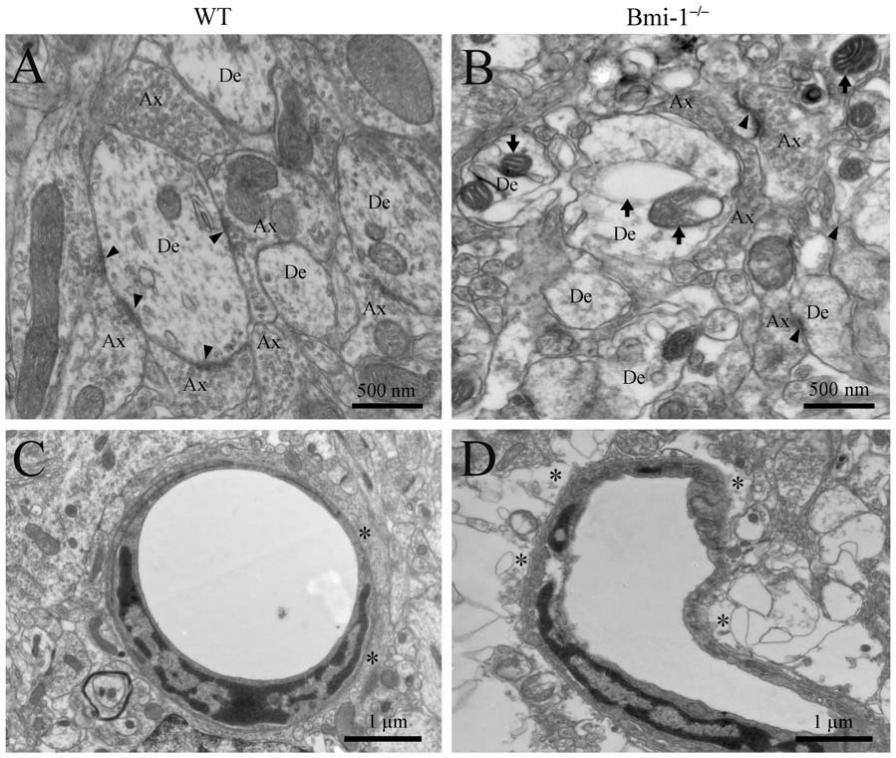
Ultrastructural alterations of the area CA1 stratum radiatum in 4-week-old Bmi-1^−/−^ mice. (**A**) A representative electron micrograph showing axonal (Ax)-dendrite (De) synapses (arrowheads) in WT mice. (**B**) Ultrastructural architecture of synaptic areas in Bmi-1^−/−^ mice. The density of synapses decreased, and the residual synapses underwent degeneration (arrowheads). Many aberrant mitochondria (arrows) exhibited “zebra-like”, swollen or vacuolar profile. In addition, the density of microtubules also decreased in the dendritic cytoplasm as compared with that in the WT littermates. (**C**) A normal brain capillary from WT mice. Note that the capillary wall was surrounded by flattened endfeet of astrocytes (stars). (**D**) Abnormal architecture of the brain capillary from Bmi-1^−/−^ mice. The capillary lumen was narrowed and obstructed accompanied with the swollen astrocyte endfeet (stars).

Ultrastructural analysis further demonstrated the occurrence of neurodegeneration in the hippocampus of Bmi-1^−/−^ mice. Swollen or vacuolar mitochondria were present at all neural elements including neuronal somas, dendrites, axonal terminals and astrocyte processes. Most of synapses underwent degeneration (Figure 3B). In addition, microvasculature exhibited considerable dilatation and distortion. Some astrocyte endfeet were highly swollen with large perivascular vacuoles (Figure 3D). These pathological alterations were not observed in WT mice (Figure 3A and C).

### Demyelination in Bmi-1 null brain

**Figure 4:**
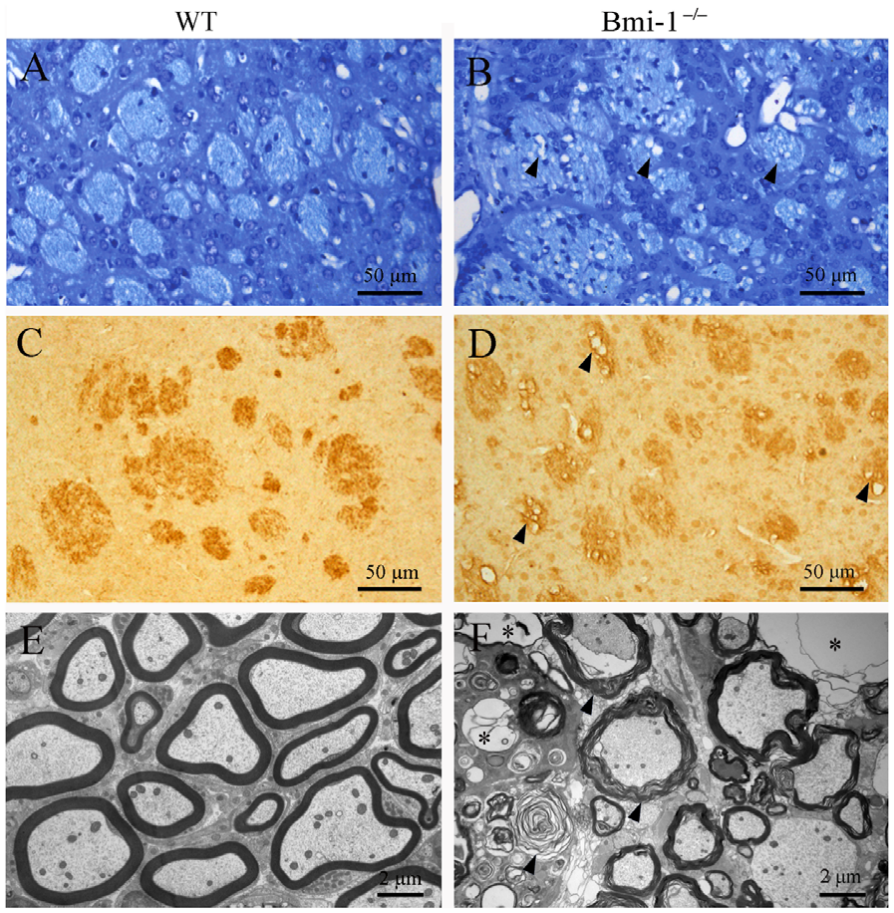
Demyelination in the striatum of 4-week-old Bmi-1^−/−^ mice. (**A–D**) Toluidine blue staining (A–B) and MBP immunohistochemistry (C–D). Many fiber bundles contained vacuoles (arrowheads) in the striatum of Bmi-1^−/−^ mice, but not in WT littermates. (**E–F**) Ultrastructure of the myelinated axons in the striatum of WT mice (E) and Bmi-1^−/−^ mice (F). Many axons underwent demyelination with stripping of the myelin lamellae (arrows in F) in Bmi-1^−/−^ mice. Some axons even totally degenerated with only empty vacuoles left.

Oligodendrocytes are also highly sensitive to oxidative stress [25]. The striatum was selected to investigate the influence of Bmi-1 deletion on axonal demyelination. Both toluidine blue histochemistry (Figure 4B) and myelin basic protein (MBP) immunohistochemistry (Figure 4D) revealed that approximately 1/3 of the fiber bundles exhibited vacuolation in the striatum of 4-week-old Bmi-1^−/−^ mice (35±4.8% vs. 0% in WT mice, n = 5 in each genotype). Electron microscopy further confirmed axonal demyelination accompanied with spongy degeneration in the Bmi-1 null striatum (Figure 4F). These pathological changes were not observed in the striatum of WT littermates (Figure 4A, C and E). Moreover, no vacuolation changes of fiber bundles were detected in the striatum of 2-week-old Bmi-1^−/−^ mice, indicating that the process of demyelination is gradual and progressive (Figure S2).

### Reactive gliosis in Bmi-1 null brain

**Figure 5:**
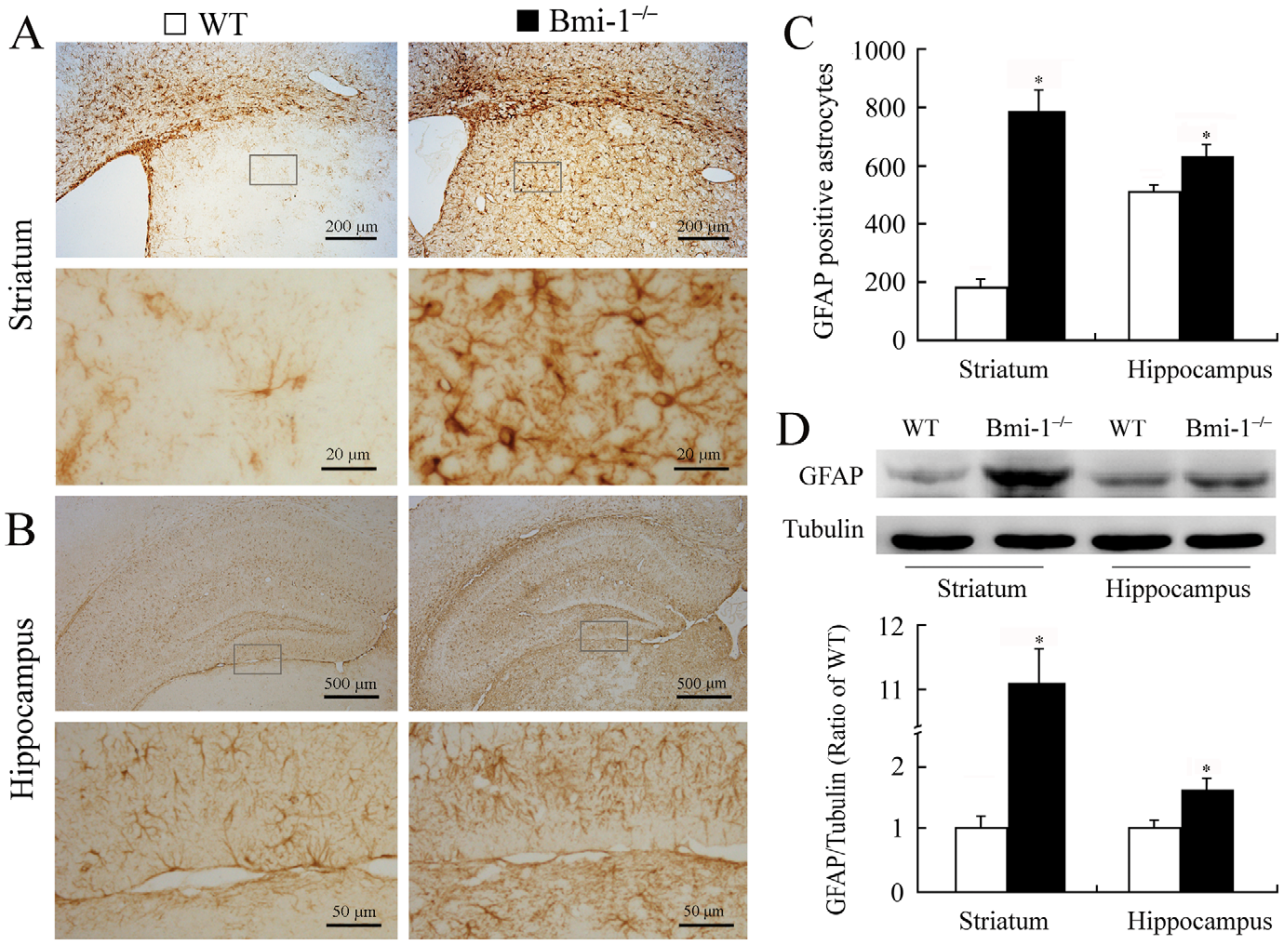
Reactive astrogliosis in the brain of 4-week-old Bmi-1^−/−^ mice. (**A–B**) Immunostaining for GFAP in the coronal sections of the striatum and hippocampus. Activated astrocytes with hypertrophic cell bodies and intensely stained processes were present in Bmi-1^−/−^ mice. (**C**) Bmi-1^−/−^ mice showed a higher number of GFAP positive astrocytes per coronal section of the striatum (786±72.4 vs. 183±28.69) and hippocampus (626±38.7 vs. 515±22.89) compared with WT controls. Five mice per genotype and 3 sections per mouse. (**D**) Bmi-1^−/−^ mice showed increases in relative protein expression levels of GFAP in the striatum (11.09±0.59 vs. 1±0.22) and hippocampus (1.63±0.22 vs. 1±0.16) compared with WT controls. Three mice per genotype and 3 independent experiments for each homogenized brain sample. Data are expressed as mean ± SEM. *P<0.05 vs. WT mice.

**Figure 6:**
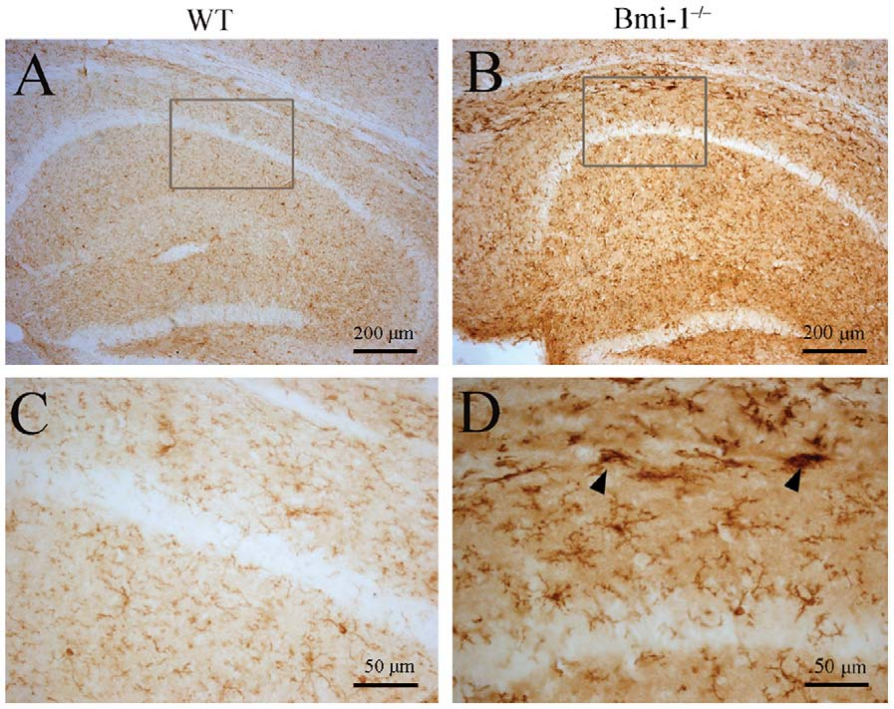
Reactive microgliosis in the hippocampus of 4-week-old Bmi-1^−/−^ mice. Compared with WT littermates (A and C), strong immunostaining for the microglial marker Iba-1 was observed in the hippocampus of Bmi-1^−/−^ mice (B and D). Activated microglia with large soma and thick processes (arrowheads) were present at Bmi-1^−/−^ mice (D).

Reactive gliosis is a pathological hallmark in neurodegenerative and demyelinating diseases [22], [26], [27]. Therefore, we observed activation of astrocytes and microglia in the striatum and hippocampus. Semi-quantitative immunohistochemistry revealed that the number of glial fibrillary acidic protein (GFAP) positive astrocytes significantly increased in the striatum and hippocampus of 4-week-old Bmi-1^−/−^ mice compared with WT controls (Figure 5A–C). Characterized by hypertrophy of cell bodies and marked upregulation of GFAP expression, a large number of activated astrocytes were observed. Consistently, Western blot analysis revealed increased expression levels of GFAP in the striatum and hippocampus of Bmi-1^−/−^ mice (Figure 5D). Similarly, immunohistochemistry for ionized calcium-binding adapter molecule (Iba-1) showed that activated microglial cells were extensively distributed in the striatum and hippocampus of Bmi-1^−/−^ mice, but not WT controls (Figure 6).

### Decreased expression of glutamate transporters and glutamine synthetase in Bmi-1 null hippocampus

**Figure 7:**
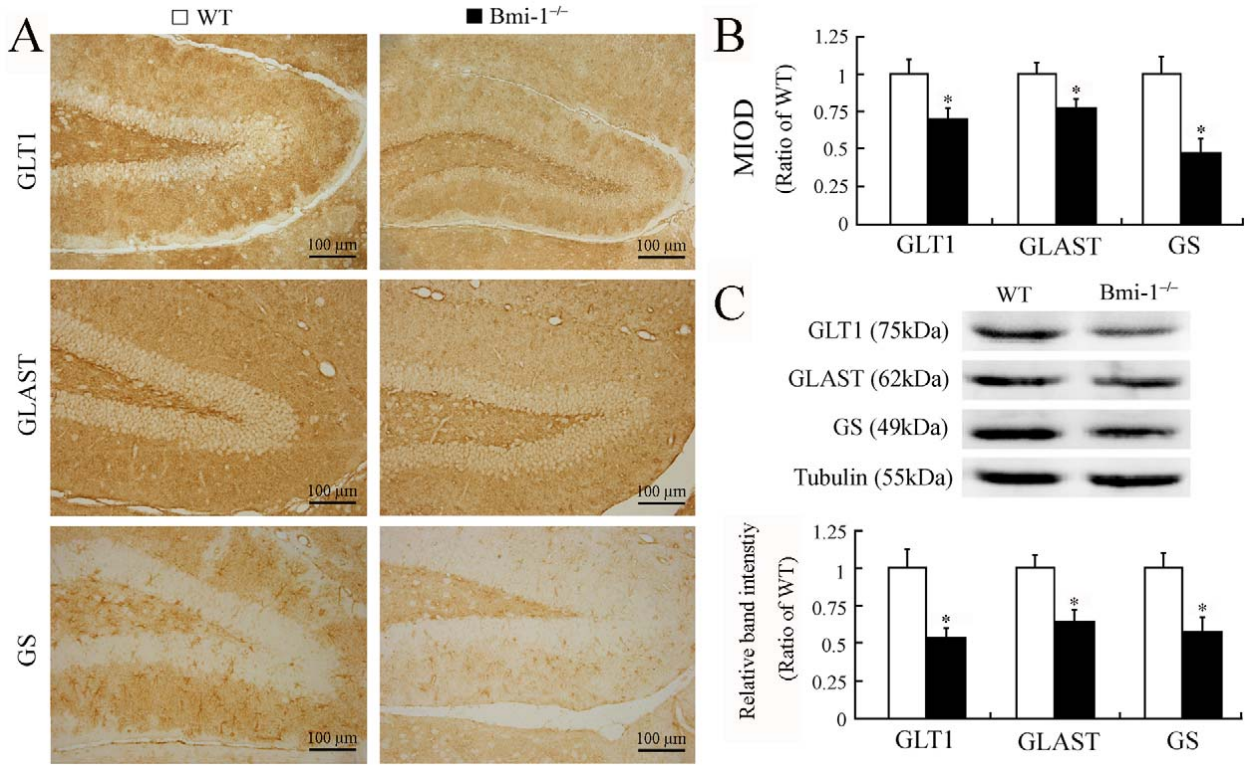
Decreased expression of GLT-1, GLAST and GS in the hippocampus of 4-week-old Bmi-1^−/−^ mice. (**A–B**) Representative micrographs showing immunoreactivities of GLT-1, GLAST and GS in the dentate gyrus of the two genotype mice. (**B**) Semi-quantitative analysis showed lower mean integrated optical densities (MIOD) of immunostainings for GLT-1 (0.71±0.07 vs. 1±0.1), GLAST (0.78±0.06 vs. 1±0.07) and GS (0.47±0.1 vs. 1±0.12) in the hippocampus of Bmi-1^−/−^ mice compared with WT controls. Five mice per genotype and 3 hippocampal sections per mouse. (**C**) Western blotting showed decreases in protein expression levels of GLT-1 (0.54±0.06 vs. 1±0.12), GLAST (0.65±0.07 vs. 1±0.08) and GS (0.58±0.1 vs. 1±0.1) in the hippocampus of Bmi-1^−/−^ mice compared with WT controls. Three mice per genotype and 3 independent experiments for each homogenized brain sample. Data represent means ± SEM. *P<0.05; vs. WT mice.

Bmi-1^−/−^ mice have sporadic epileptic-like seizures and tremors, but the underlying mechanisms need further investigation [16], [17]. Emerging evidence reveals a critical role of astrocyte dysfunction, including altered glutamate uptake and conversion in the pathogenesis of epilepsy [23], [24]. Thus, we investigated the expression levels of glutamate transporter 1 (GLT-1), glutamate/aspartate transporter (GLAST) and glutamine synthetase (GS) in the hippocampus, a key region associated with seizure occurrence in rodents. Four-week-old Bmi-1^−/−^ mice showed decreased GLT-1, GLAST and GS protein levels, as revealed by both immunohistochemistry (Figure 7A–B) and Western blot analysis (Figure 7C). Supplemental data revealed that these markers were not altered in the hippocampus of 2-week-old Bmi-1^−/−^ mice compared with WT littermates, indicating that glutamate uptake and conversion impairment is due to oxidative stress rather than retardation in astrocyte maturation (Figure S3).

## Discussion

Bmi-1 is essential for the maintenance and self-renewal of both hematopoietic and neural stem cells, primarily via the Ink4a locus encoding p16Ink4a/p19Arf [10], [11], [12], [13], [14], [15], [28], [29], [30], [31]. However, deletion of both Ink4a and Arf fails to rescue the growth retardation or improve overall survival of Bmi-1^−/−^ mice, indicating that additional Bmi-1-regulated pathways may exist [15], [32], [33]. Furthermore, evidence from recent literature suggests that Bmi-1 is necessary for cellular oxidative metabolism. Liu and colleagues (2009) have demonstrated that Bmi-1 regulates mitochondrial function, ROS levels and the DNA damage response pathway in cultured bone marrow cells and in freshly isolated thymocytes, independent of the Ink4a/Arf pathway [7]. Treatment with the antioxidant N-acetylcysteine or disruption of the DNA damage response pathway by Chk2 deletion can partially rescue various abnormalities including the neurological defects in Bmi-1^−/−^ mice [7]. Another recent study has also revealed that Bmi-1 regulates antioxidant defenses in cultured cortical neurons by repressing p53 pro-oxidant activity [8]. The present results have shown that Bmi-1 absence results in brain oxidative damage during the postnatal development. This finding provides insight into the function of Bmi-1 in regulating brain antioxidant defense and controlling of the normal lifespan of brain cells.

Pathological analysis on the hippocampus of 4-week-old Bmi-1^−/−^ mice indicates that deterioration of neurons is more prominent on axonal terminals than cell bodies. This pattern of neuronal degeneration is consistent with the viewpoint that long, extended axons and their synapses seem to be more vulnerable in an oxidative environment due to a high metabolic rate and low antioxidant defenses at synaptic terminals compared with neuronal soma [34]. Oxidative stress also plays a critical role in demyelinating diseases such as multiple sclerosis [25]. We found that absence of Bmi-1 causes extensive demyelination in the striatum at 4 weeks after birth. The widespread degeneration and demyelination leads to severe reactive gliosis, which in turn may produce a large amount of ROS [22]. This vicious cycle exacerbates the Bmi-1 absence-induced oxidative damage.

In contrast to neurons and oligodendrocytes undergoing cellular loss or degeneration, astrocytes exhibit activation and proliferation, termed “reactive astrogliosis”, in response to a variety of CNS injuries including oxidative damage [22]. We have demonstrated that reactive astrogliosis occurs in 4-week-old Bmi-1^−/−^ brain and is more serious in the white matter than in the gray matter. Furthermore, apart from increased GFAP expression, alterations of astrocyte markers including GLT1, GLAST and GS are observed, but these changes have not detected at 2 weeks after birth. These results suggest that reactive astrogliosis is neither an all-or-none response nor a single uniform process. Instead, reactive astrogliosis is a finely gradated continuum of progressive changes in gene expression and cellular morphology [22].

Astrocytes are more resistant to oxidative damage than neurons and oligodendrocytes because they contain a variety of antioxidants, vitamins and oxidative defense enzymes [35], [36], [37]. However, sustained oxidative stress also impairs cellular structures and functions of astrocytes [38]. Glutamate transporters and GS seem to be highly susceptible to oxidative damage [39], [40], [41]. Decreased expressions of glutamate transporters and GS have been observed in postmortem hippocampal tissues from epileptic patients, as well as from animal models of epilepsy [42], [43], [44], [45], [46]. Thus, decreased GS, GLT1 and GLAST would hamper glutamate clearance and conversion, subsequently exacerbating glutamate-mediated neurotoxicity. This may represent a plausible mechanism for sporadic epileptic-like seizures in Bmi-1^−/−^ mice.

Additionally, impaired glutamate conversion in astrocyte processes would result in intracellular hyperosmotic pressure, thus drawing additional water into the astrocytes and causing their perivascular endfeet to swell, as demonstrated by electron microscopy. Astrocyte endfeet, one component of the blood-brain barrier, serve as the first line of defense in response to a variety of toxic agents in the blood stream [21]. Highly swollen endfeet may fail to protect the brain parenchyma from toxic substances found in the circulatory system, which in turn exacerbates neuropathological impairment in Bmi-1^−/−^ brain.

In conclusion, we have demonstrated that the polycomb group protein Bmi-1 is necessary for regulating endogenous antioxidant defenses in the brain, and its absence causes oxidative damage in the premature mouse brain. Bmi-1^−/−^ mice may serve as a suitable model for preclinical evaluation of the neuroprotective efficacy of antioxidant agents.

## Materials and Methods

### Mice and genotyping

Bmi-1^+/−^ mice (129Ola/FVB/N hybrid background) that were backcrossed 10–12 times onto a C57BL/6J background were mated to generate 2 or 4-week-old (postnatal 14 or 28-day-old) Bmi-1^−/−^ mice and WT littermates genotyped by PCR, as described previously [33]. This study was carried out in strict accordance with international standards on animal welfare and the guidelines of the Institute for Laboratory Animal Research of Nanjing Medical University. The protocol was approved by the Committee on the Ethics of Animal Experiments of Nanjing Medical University (Permit Number: BK2006576). All efforts were made to minimize animal suffering and to reduce the number of animals used.

### Preparation of brain sections

The mice were anesthetized with sodium pentobarbital and transcardially perfused with 0.9% saline followed by 1% paraformaldehyde with 1% glutaraldehyde (for conventional electron microscopy) or 4% paraformaldehyde (for histochemistry or immunohistochemistry) in phosphate buffer (PB, 0.1 M, pH 7.4). The brains were dissected and postfixed overnight at 4°C. For electron microscopy, 80 µm forebrain or hippocampus cross-sections were sliced via a vibratome. The striatum and CA1 stratum radiatum were trimmed, dehydrated and embedded in Epon 812. Ultrathin sections of 70 nm were obtained, counterstained on copper grids with both uranyl acetate and lead citrate, and examined with a Jeol 1200EX electron microscope (Tokyo, Japan). For histochemistry or immunohistochemistry, some sections were dehydrated in a series of graded ethanol solutions and embedded in paraffin. Next, the coronal sections of the forebrain or hippocampus were cut to thickness of 5 µm and divided into six series. The remaining brain tissues were stored in 30% sucrose solution at 4°C for 48 h, and cut transversely on a freezing microtome at 40 µm thickness (Leica, Nussloch, Germany). The sections were collected in six series and used for Iba-1 immunostaining.

### Toluidine blue staining

Paraffin-bedded forebrain sections were stained in 0.5% toluidine blue for 10 minutes after dewaxing and rehydration. Following a distilled water wash, the sections were dehydrated through a graded series of alcohol and vitrificated in xylene.

### Immunohistochemical staining

The protocol for immunohistochemisty was as described previously [47]. Briefly, sections were incubated with the primary antibody as following: mouse anti-GFAP (1∶1500, Sigma-Aldrich, Saint Louis, MO, USA), rabbit anti-caspase-3 (1∶1500, Millipore, Billerica, MA, USA), guinea pig anti-GLT-1 (1∶1000, Millipore), guinea pig anti-GLAST (1∶1000, Millipore), rabbit anti-GS (1∶200, Santa Cruz BioTech, Santa Cruz, CA, USA), rabbit anti-Iba-1 (1∶500, Wako, Wako, Osaka, Japan), mouse anti-synaptophysin (1∶1200, Sigma-Aldrich) or mouse anti-β-tubulin III (1∶2000, Sigma-Aldrich) at 4°C overnight. After rinsing in PBS, the sections were incubated with biotinylated goat anti-rabbit (1∶200), mouse (1∶200) or guinea pig (1∶200) IgG for 1 h at room temperature and visualized using Elite ABC Kit (Vector, Burlingame, CA, USA).

### Quantitative analysis of immunostaining

The number of GFAP positive astrocytes per striatum or hippocampus cross-section was counted at a magnification of ×100 using a digital microscope (Leica Microsystems, Wetzlar, Germany). Three sections per mouse, and five mice per genotype, were averaged to provide a mean value for each group. The number of caspase-3 positive hippocampal neurons was also counted by the same method. In addition, the extent of demyelination within striatum was assessed on coronal sections stained with Toluidine blue or MBP. The ratio of the fiber bundles containing vacuoles to the total fiber bundles per striatum section was analyzed. Finally, the mean integrated optical density (MIOD) was measured to assess the expression levels of synaptophysin, β-tubulin III, GLT-1, GLAST, and GS in the whole hippocampus regions at a magnification of ×100, respectively, using an Image-Pro Plus 6.0 Analysis System (Media Cybernetics Inc., San Francisco, CA, USA) [47].

### Western blotting

Proteins were extracted from the hippocampus or striatum and quantified with a kit (Bio-Rad, Mississauga, Ontario, Canada). Ten µg protein samples were fractionated by SDS-PAGE and transferred to nitrocellulose membranes. The immunoblotting was carried out as described using antibodies against with GFAP (1∶2000), GLT-1 (1∶1000), GLAST (1∶1000) and GS (1∶200) [47]. The immunocomplexes were visualized using the ECL detection kit (Amersham Pharmacia Biotech, Canada). Membranes were scanned and analyzed using an Omega 16ic Chemiluminescence Imaging System (Ultra-Lum, USA).

### Biochemical measurement

The brain tissues were homogenized in cold saline. The homogenate (10%) was centrifuged at 4000 *rpm* at 4°C for 10 min. The supernatant was used for measurements of hydroxyl radical, malondialdehyde and protein carbonyl levels. All examinations were performed according to the manufacturer's instructions (Jiancheng Institute of Biotechnology, China). The detailed methods have been described in the previously published report [48].

### Statistical Analysis

Data are presented as the mean ± SEM. Statistical comparisons were made using the Student's t-test, with a probability value <0.05 being considered significant.

## Supporting Information

Figure S1 **Oxidative parameters in 2-week-old Bmi-1^−/−^ mice and wild-type controls.** Brain tissues from Bmi-1^−/−^ mice showed higher levels of hydroxyl radical (93.82±7.54 vs. 62.81±6.35 U/mg protien; A), protein carbonyl (3.73±0.25 vs. 3.47±0.22 nmol/mg protein; B) and malondialdehyde (3.22±0.25 vs. 2.99±0.23 nmol/mg protein; C) than those from WT controls, but the significant difference was only hydroxyl radical levels. Five mice per genotype and 3 independent experiments for each homogenized brain sample. Data are expressed as mean ± SEM. *P<0.05 vs. WT mice.
(TIF)

Figure S2 **Immunohistochemistry for MBP in the striatum of 2-week-old Bmi-1^−/−^ mice and wild-type controls.** (**A**–**D**) The distributional pattern of MBP positive fibers bundles within the striatum was similar between the two genotype mice, although MBP immunostaining was weaker in Bmi-1^−/−^ mice.
(TIF)

Figure S3 **Expression of GLT-1, GLAST and GS in the hippocampus of 2-week-old Bmi-1^−/−^ mice and wild-type controls.** (**A**) Representative micrographs showing immunoreactivities of GLT-1, GLAST and GS in the hippocampal CA1 region of the two genotype mice. (**B**) Semi-quantitative analysis showed that there were no significant differences in the mean integrated optical densities (MIOD) of immunostainings for GLT-1 (0.88±0.15 vs. 1±0.12), GLAST (0.94±0.1 vs. 1±0.11) and GS (1.04±0.16 vs. 1±0.12) in the hippocampus of Bmi-1^−/−^ mice and WT controls. Five mice per genotype and 3 hippocampal sections per mouse. (**C**) There were no significant differences in protein expression levels of GLT-1 (0.94±0.14 vs. 1±0.12), GLAST (0.9±0.15 vs. 1±0.11) and GS (0.97±0.11 vs. 1±0.07) in the hippocampus of Bmi-1^−/−^ mice and WT controls. Three mice per genotype and 3 independent experiments for each homogenized brain sample. Data represent means ± SEM. *P<0.05 vs. WT mice.
(TIF)
