# Correction: Bmi-1 Absence Causes Premature Brain Degeneration

**DOI:** 10.1371/annotation/1f96f6db-9756-4eee-9ca5-29a194516894

**Published:** 2012-07-10

**Authors:** Guangliang Cao, Minxia Gu, Min Zhu, Junying Gao, Ying Yin, Charles Marshall, Ming Xiao, Jiong Ding, Dengshun Miao

There was an error in the Author Contributions section. The second contributions category should read: "Performed the experiments: GC MG MZ; GC and MG contributed equally to this work." 

