# Supplementary figures and images for: Bmi-1 Absence Causes Premature Brain Degeneration
Source: PLoS One. 2012 Feb 20;7(2):e32015. doi: 10.1371/journal.pone.0032015 (PMC3282795; doi:10.1371/journal.pone.0032015)

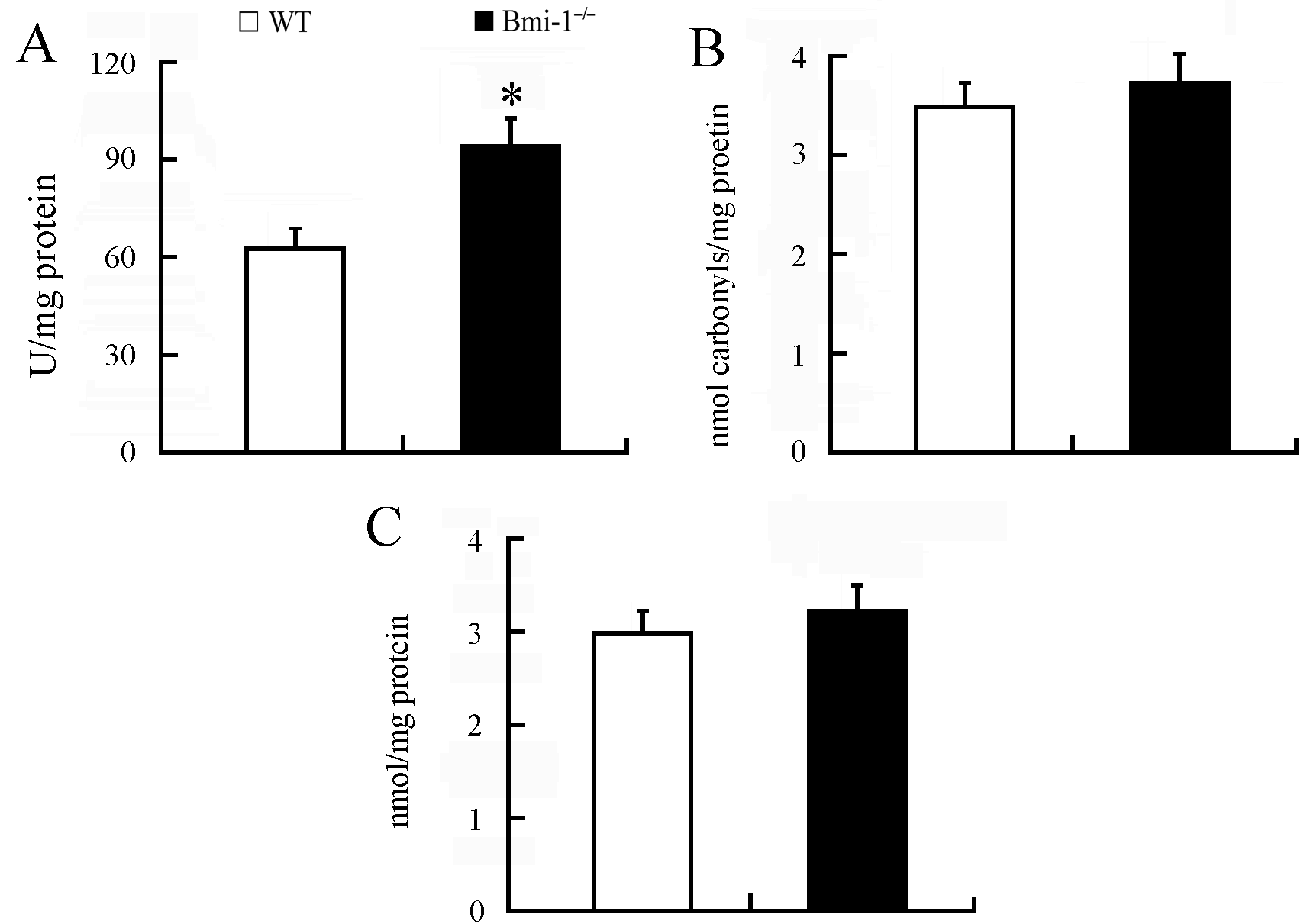

Supplement: Figure S1 — Oxidative parameters in 2-week-old Bmi-1−/− mice and wild-type controls. Brain tissues from Bmi-1−/− mice showed higher levels of hydroxyl radical (93.82±7.54 vs. 62.81±6.35 U/mg protien; A), protein carbonyl (3.73±0.25 vs. 3.47±0.22 nmol/mg protein; B) and malondialdehyde (3.22±0.25 vs. 2.99±0.23 nmol/mg protein; C) than those from WT controls, but the significant difference was only hydroxyl radical levels. Five mice per genotype and 3 independent experiments for each homogenized brain sample. Data are expressed as mean ± SEM. *P<0.05 vs. WT mice. (TIF) [file pone.0032015.s001.tif]

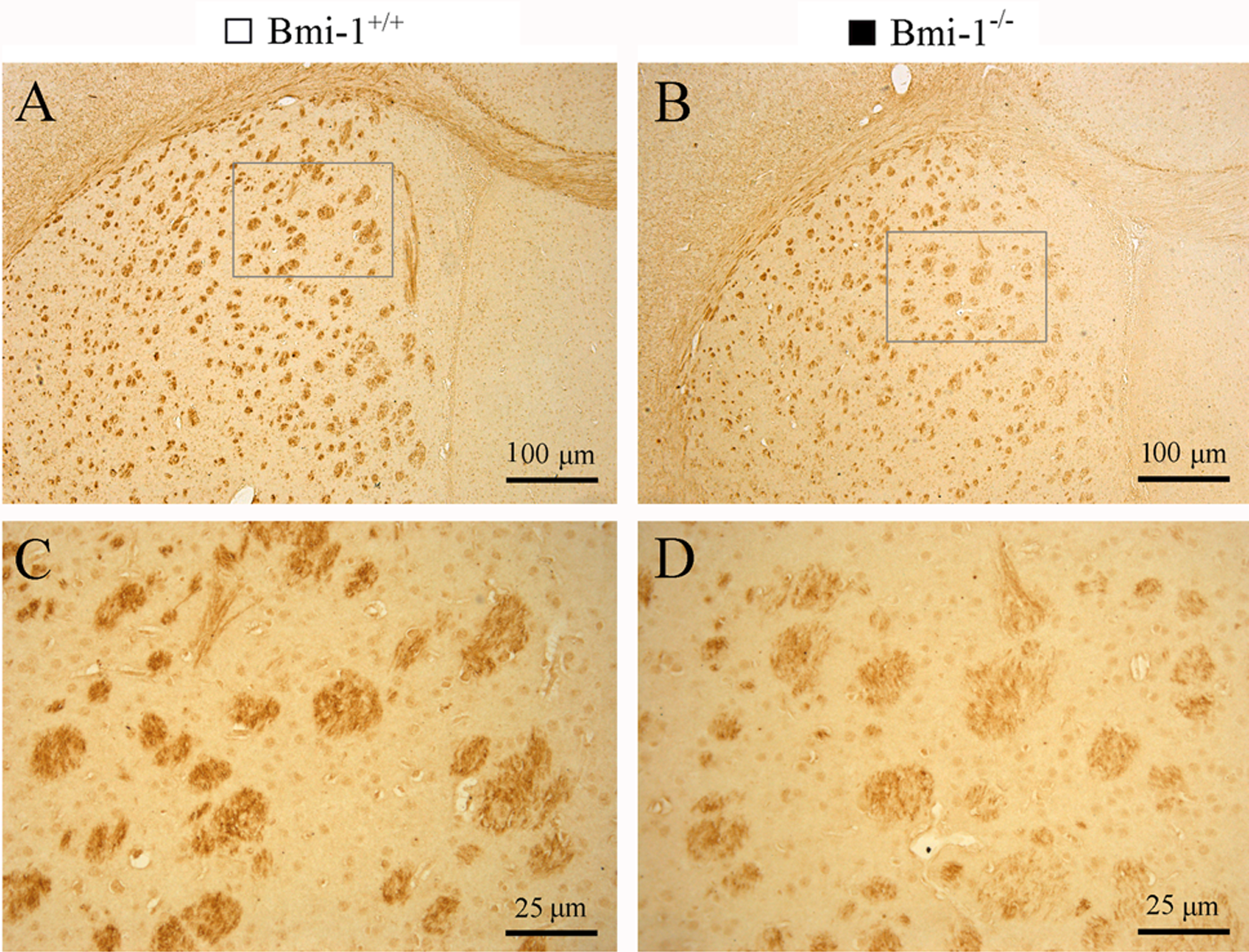

Supplement: Figure S2 — Immunohistochemistry for MBP in the striatum of 2-week-old Bmi-1−/− mice and wild-type controls. (A–D) The distributional pattern of MBP positive fibers bundles within the striatum was similar between the two genotype mice, although MBP immunostaining was weaker in Bmi-1−/− mice. (TIF) [file pone.0032015.s002.tif]

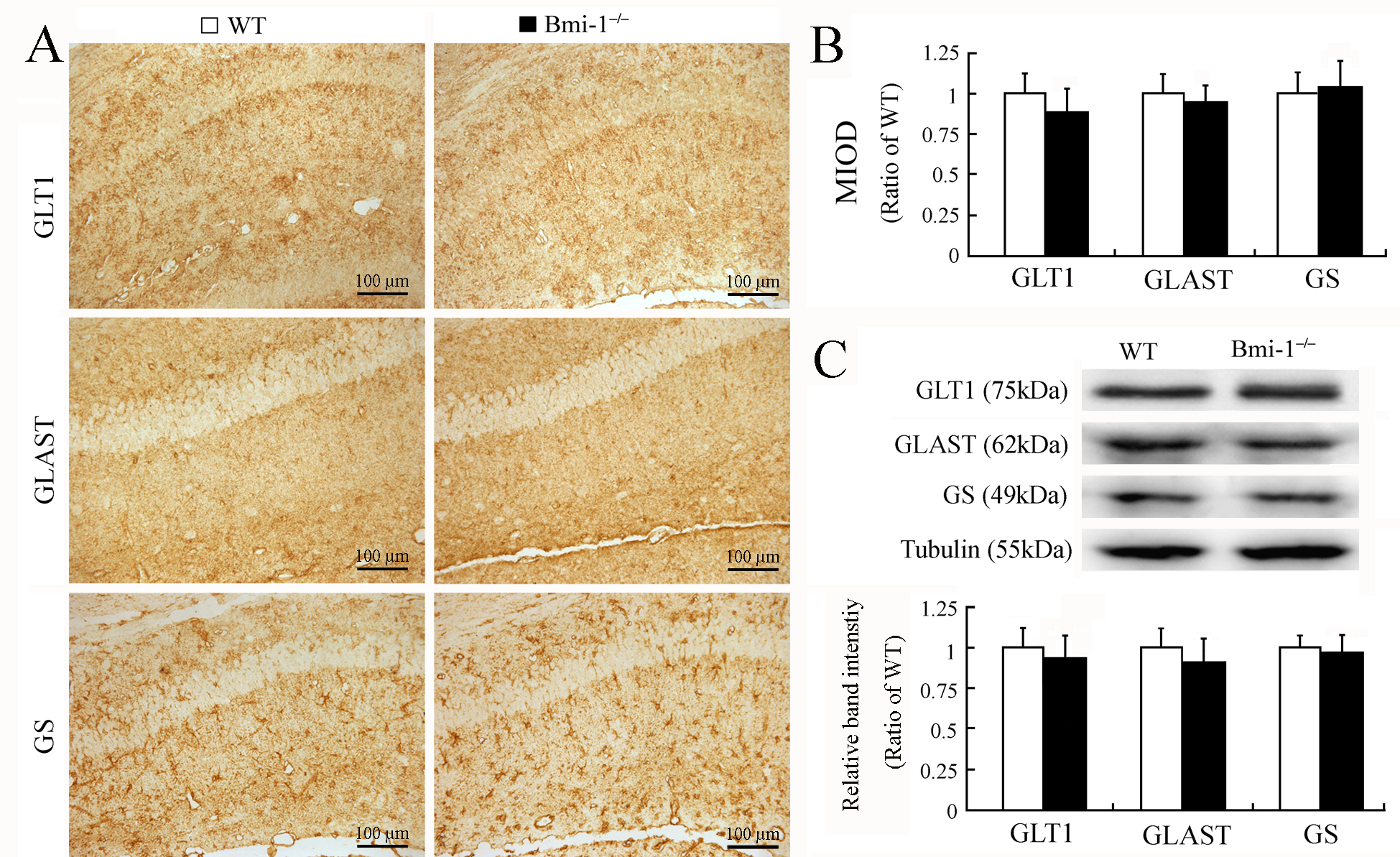

Supplement: Figure S3 — Expression of GLT-1, GLAST and GS in the hippocampus of 2-week-old Bmi-1−/− mice and wild-type controls. (A) Representative micrographs showing immunoreactivities of GLT-1, GLAST and GS in the hippocampal CA1 region of the two genotype mice. (B) Semi-quantitative analysis showed that there were no significant differences in the mean integrated optical densities (MIOD) of immunostainings for GLT-1 (0.88±0.15 vs. 1±0.12), GLAST (0.94±0.1 vs. 1±0.11) and GS (1.04±0.16 vs. 1±0.12) in the hippocampus of Bmi-1−/− mice and WT controls. Five mice per genotype and 3 hippocampal sections per mouse. (C) There were no significant differences in protein expression levels of GLT-1 (0.94±0.14 vs. 1±0.12), GLAST (0.9±0.15 vs. 1±0.11) and GS (0.97±0.11 vs. 1±0.07) in the hippocampus of Bmi-1−/− mice and WT controls. Three mice per genotype and 3 independent experiments for each homogenized brain sample. Data represent means ± SEM. *P<0.05 vs. WT mice. (TIF) [file pone.0032015.s003.tif]
